# Serous endometrial carcinoma metastatic to the sigmoid colon masquerading as a primary colon cancer detected by bowel obstruction

**DOI:** 10.1186/s40792-024-02073-8

**Published:** 2024-11-28

**Authors:** Taiga Shimura, Naoko Fukushima, Kazuto Tsuboi, Toshimasa Suzuki, Tetsuya Kajimoto

**Affiliations:** 1 Department of Surgery, Fuji General Hospital, Shizuoka, Japan; 2 Department of Surgery, The Jikei University School of Medicine, Tokyo, Japan

**Keywords:** Endometrial carcinoma, Colon metastasis, Colon cancer, Metal stent

## Abstract

**Background:**

The majority of colorectal malignancies are primary tumors. Secondary tumors are rare, and colorectal metastasis from endometrial carcinoma is exceptionally uncommon. We report a case of serous endometrial carcinoma that metastasized to the sigmoid colon, initially presenting as a primary colon carcinoma due to bowel obstruction.

**Case presentation:**

A 79-year-old woman presented with abdominal distention and constipation. Five years earlier, she had undergone an open total hysterectomy with bilateral salpingo-oophorectomy for endometrial serous carcinoma. A colonoscopy revealed stenosis encircling the entire sigmoid colon. Abdominal computed tomography demonstrated irregular wall thickening in the sigmoid colon with enhanced regional lymph node enlargement and upstream bowel dilatation. Peritoneal dissemination in the pelvis was also noted. To relieve the obstruction, two self-expanding metal stents were inserted. Subsequently, due to the presumed diagnosis of sigmoid colon carcinoma with peritoneal dissemination, an open left hemicolectomy with resection of the peritoneal dissemination was performed. Histopathological examination identified the colon tumor and peritoneal dissemination as metastatic serous endometrial carcinoma. Immunohistochemical studies showed the tumor cells were negative for CK7, CK20, and CDX2. No chemotherapy was administered, and 6 months post-operation, no recurrence was observed.

**Conclusions:**

Metastasis of endometrial carcinoma to the colon is extremely rare. Diagnosing a colon tumor as a metastasis from endometrial carcinoma is challenging during preoperative examinations. Therefore, in patients with a history of endometrial carcinoma, the possibility that the primary site might be the uterus should be considered.

## Background

The majority of colorectal tumors are primary in nature, with metastatic tumors being less common. Among these, colorectal metastasis from endometrial carcinoma (EC) is extremely rare and challenging to distinguish from primary colorectal cancer. In this report, we describe a case of endometrial serous carcinoma that metastasized to the sigmoid colon, presenting as a primary colon cancer detected due to bowel obstruction.

## Case report

**Fig. 1:**
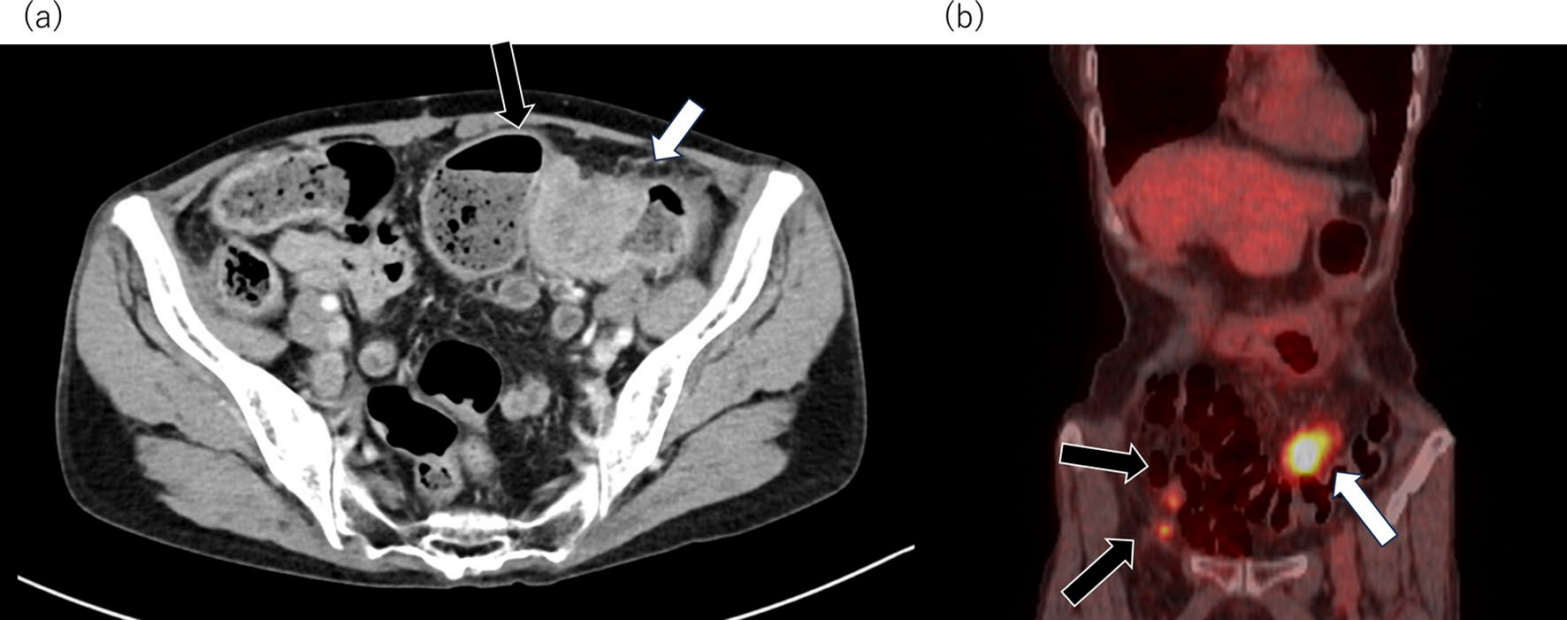
**a** Enhanced abdominal computed tomography showed irregularly enhanced wall thickening in the sigmoid colon (white arrow). The tumor was in contact with the transverse colon (black arrow). **b** Positron emission tomography (PET)–CT demonstrated accumulation in sigmoid colon tumor (SUVmax: 9.49, white arrow), and two peritoneal disseminations in the pelvis (SUVmax: 3.85, black arrow)

**Fig. 2:**
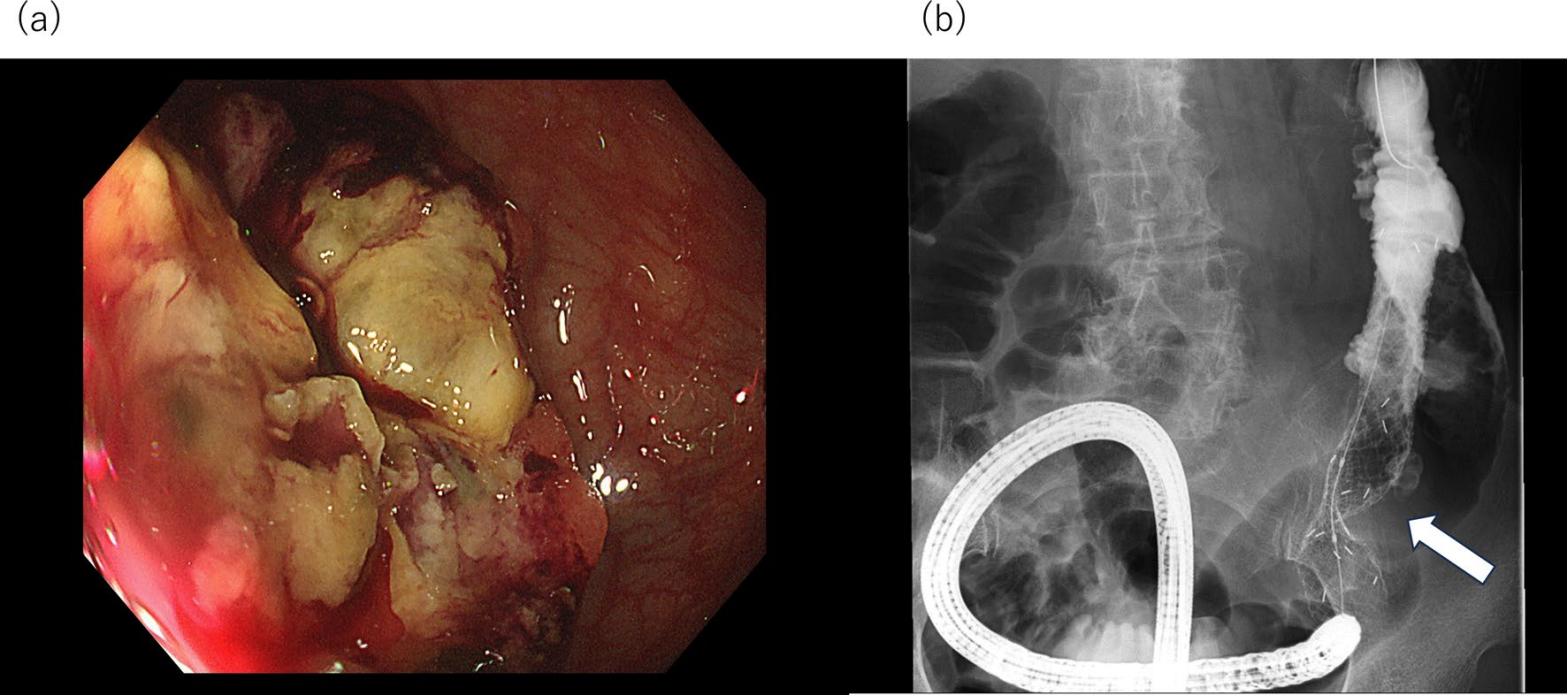
**a** Colonoscopy showed stenosis over the entire circumference of sigmoid colon, b two self-expanding metal stents were placed to span the point of the obstruction (white arrow)

**Fig. 3:**
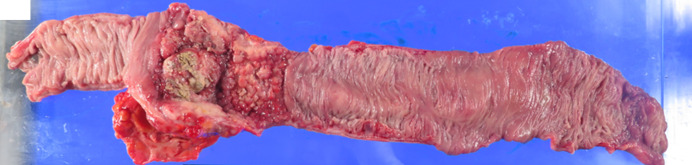
Resected specimen

**Fig. 4:**
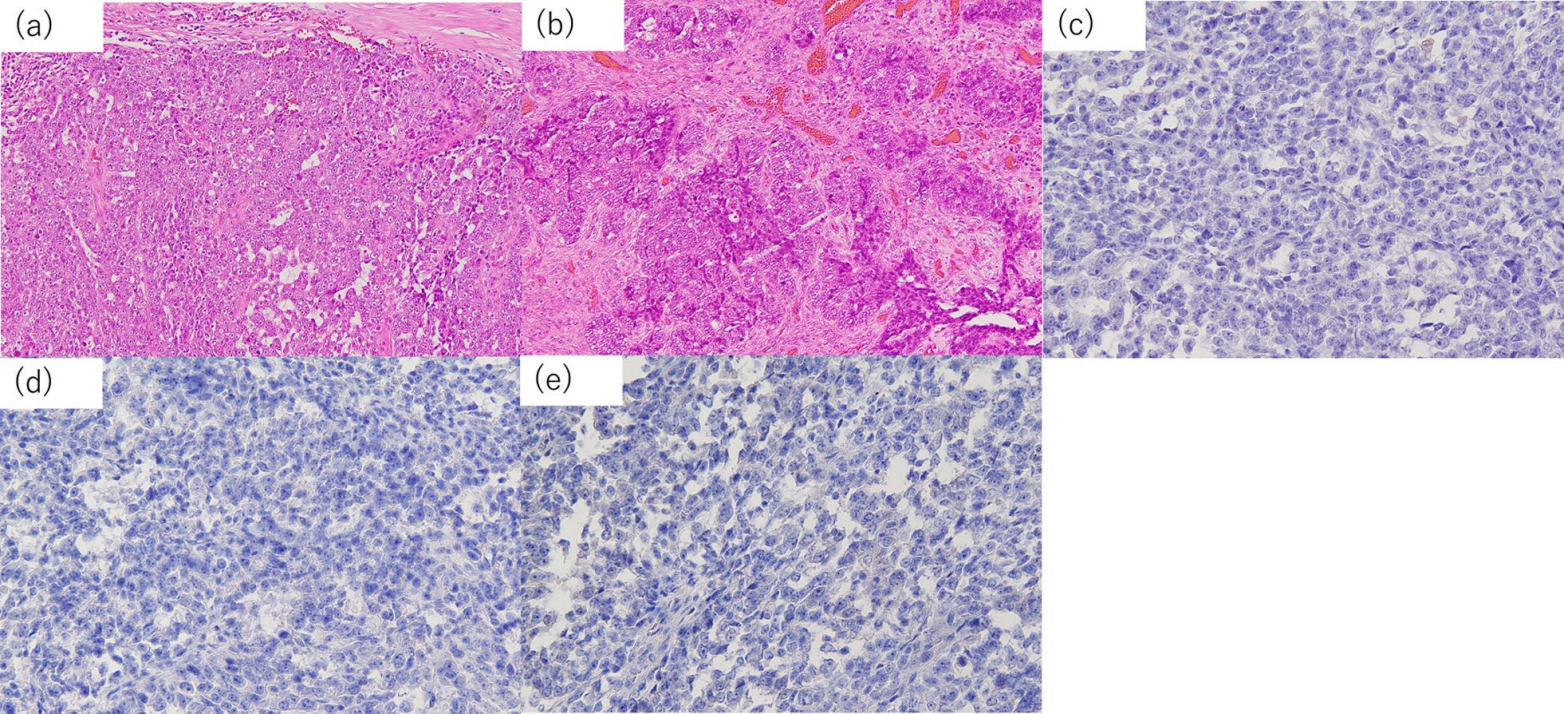
Histopathological examination showed endometrial serous carcinoma of colon tumor (**a**) and peritoneal disseminations in pelvis (**b**) (×100). Immunostaining was negative for CK7 (**c**), CK20 (**d**), and CDX2 (**e**) (×100)

A 79-year-old woman presented with abdominal distention and constipation. Five years earlier, she had undergone an open total hysterectomy with bilateral salpingo-oophorectomy for endometrial serous carcinoma at our hospital (T1aN0M0, Stage IA with lymph vascular invasion, according to the 3rd edition of The General Rules for Clinical and Pathological Management of Uterine Cancer). The patient had not returned for follow-up after surgery due to self-interruption. During the physical examination, abdominal tenderness was noted. Laboratory tests showed an elevated CRP level of 7.9 mg/dL, but other results, including tumor markers (carcinoembryonic antigen: 3.4 ng/mL, carbohydrate antigen 19–9: 39.2 U/mL), were within normal limits. Enhanced computed tomography (CT) revealed irregularly enhanced wall thickening in the sigmoid colon with regional lymph node enlargement and oral-side bowel dilatation (Fig. 1a). Positron emission tomography (PET)–CT demonstrated accumulation in the pelvis, suggesting peritoneal dissemination (Fig. 1b). No liver or lung metastases were detected. A colonoscopy showed stenosis encompassing the entire circumference of the sigmoid colon (Fig. 2a). To alleviate the bowel obstruction, two self-expanding metal stents (Niti-S uncovered stent, Taewoong Medical, Gimpo, Korea, sizes 22 × 120 mm and 22 × 60 mm) were placed across the obstruction (Fig. 2b). The tumor had a long diameter of 95 mm, and an additional 60 mm stent was placed after the 120-mm stent placement to obtain adequate stent dilation. Biopsy revealed a moderately differentiated tubular adenocarcinoma. The diagnosis was colon cancer cT3N0M1c Stage IVc according to the Japanese Classification of Colorectal, Appendiceal, and Anal Carcinoma. Complete resection was deemed feasible, and two months later for patient’ convenience, an open left hemicolectomy with D3 lymph node dissection was performed. The tumor was adherent to the transverse colon and was easily detached, however, invasion could not be ruled out completely. Therefore, the transverse colon warranted its inclusion in the resection. Retroperitoneal invasion was suspected and thus addressed during surgery. Four peritoneal disseminations in the omentum and pelvis were identified and resected. Macroscopically, a 95 mm × 90 mm tumor with an implanted stent was found in the sigmoid colon (Fig. 3). Histopathological examination revealed endometrial serous carcinoma in the colon tumor (Fig. 4a) and peritoneal disseminations in the omentum and pelvis (Fig. 4b). The tumor had invaded the retroperitoneum but was completely resected, with no invasion into the transverse colon observed. All microscopic margins were clear. Vessel and lymph duct invasion were noted in the lesion. No cancer metastasis was found in the 18 dissected regional lymph nodes. Immunohistochemical results showed the tumor cells were negative for CK7, CK20, and CDX2 (Fig. 4c–e). Thus, the pathological diagnosis confirmed the metastasis of endometrial serous carcinoma in the colon and peritoneal metastasis. The patient was discharged 9 days postoperatively without complications. No chemotherapy was administered, and 6 months post-operation, no recurrence was observed.

## Discussion

EC is the fourth most common type of cancer among women, and its mortality rate has risen over the past 20 years [1]. Endometrioid carcinoma is the most prevalent histological type, while serous endometrial carcinoma, which comprises 5–10% of all EC cases [2], carries a poorer prognosis, with a 5-year survival rate between 60 and 65% [3]. EC metastasis primarily occurs in the vagina (42%), lungs (27%), and peritoneum (27%) [1]. Metastasis to the colon is extremely rare and is most often attributed to direct spread or peritoneal dissemination. In addition, it has been suggested that EC can also disseminate transovarially, even in the early stages [4]. Atypical metastatic sites for EC, such as those associated with a history of endometriosis, have been reported [5]. However, in the current case, there is no history of endometriosis or symptoms suggesting such a condition. While the prognosis in the early stages is generally favorable, with infrequent local or distant recurrences, metastases can manifest in various locations years after the initial treatment [6]. Risk factors for recurrence in early-stage EC include a histologic grade 3, age > 60 years, depth of myometrial invasion, lymph vascular invasion, and involvement of the lower uterus [7]. In this case, the patient’s age and lymph vascular invasion were significant risk factors.

Most colon carcinomas are primary tumors; however, secondary tumors can also be found. When comparing the frequency of primary sites for metastatic colon tumors, the stomach and ovaries are the most common, followed by the pancreas [8]. The uterine endometrium is considered a relatively rare primary site. Endometrial and colorectal carcinomas exhibit morphological similarities, often causing colon tumors to be mistaken for primary carcinomas. In the current case, a preoperative endoscopic biopsy indicated a diagnosis of colorectal carcinoma, leading to surgery under the suspicion of this diagnosis. However, distinct immunohistochemical profiles can differentiate between these cancers. It has been reported that 75–95% of carcinomas originating from the colonic mucosa are CK7 negative and CK20 positive, whereas 80–100% of carcinomas originating from endometriosis are CK7 positive and CK20 negative [9, 10]. Additionally, CDX2 is a potential marker for primary colorectal tumors, with most studies showing that CDX2 is a sensitive marker with a reported sensitivity of up to 90% [11]. In the current study, both CK7 and CK20 were negative, but CDX2 was also negative, and the histological diagnosis was serous carcinoma consistent with endometrial carcinoma tissue, leading to the diagnosis of EC metastasis. Metastatic forms include lymph vascular and disseminated, and it is difficult to conclude that this is metastasis to the colon rather than the invasion of peritoneal dissemination. The classification of gross appearance is often described as ulcer localized type in the lymph vascular form and ulcer invasion type in the disseminated form [12]. In the current case, the classification of gross appearance is ulcer localized type. In addition, the main site of tumor was not on the serosa, but on the submucosa and muscle layers which is common in the lymph vascular form [12]. Therefore, we thought this case was most likely metastasis to the colon rather than the invasion of peritoneal dissemination.

Colorectal metastases, similar to primary colorectal carcinoma, are often detected through symptoms such as abdominal pain or bleeding. In the present case, the patient presented with abdominal pain and intestinal obstruction. However, there is only one previously reported case where the patient presented with intestinal obstruction due to colorectal metastasis [13]. For the treatment of EC metastasis, tumor resection or tumor reduction surgery is recommended [14]. Given the patient’s bowel obstruction, a stent was inserted preoperatively, followed by a standby operation. To the best of our knowledge, this is the first reported case of bowel obstruction due to EC metastasis where a stent was inserted, and the patient was operated on standby. Postoperative chemotherapy options include doxorubicin + cisplatin or paclitaxel + carboplatin therapy. In this case, chemotherapy was not selected, considering the patient’s preference.

There have been 9 [6–9, 13, 15–18] reported cases of colonic metastasis of EC, with 10 cases, including ours, listed in Table 1. Endometrioid carcinoma was the most common type, with only two cases being serous carcinoma, like the present case. The stages at initial surgery were Stage I in 5 cases, Stage II in 1 case, Stage III in 2 cases, and 2 cases with uncertain staging. This indicates that colon metastasis can occur even at an early stage. Additionally, there is a wide range in the interval between the first surgery and recurrence (0.1–15 years), with the longest interval being 15 years, suggesting that recurrence may occur several years later. Preoperative diagnosis of colon metastasis of EC was possible in 5 cases, while 4 cases, including ours, were initially diagnosed as primary colon tumors. Therefore, clinicians must consider the possibility of metastatic disease, especially if there is a history of EC. Furthermore, follow-up is crucial because recurrence can occur several years later, even in early-stage cancer.

**Table 1:** Clinicopathologic findings of endometrial carcinoma metastatic to colon or rectum

Author	Year	Age (year)	Interval (year)	Stage	Pathology	Chemo/radiation (after first operation)	Symptoms	Location	Size (cm)	Surgery	Chemo/radiation (after second operation)	Endometriosis	Outcome (month)
Our case	2024	79	5	IA	Serous ca	−	Abdominal pain, ileus	S, peritoneum	9	LHC	−	−	6 alive
Cao [13]	2023	68	2	N/A	Endometrioid ca	−	Ileus	T	2.4	N/A	N/A	N/A	N/A
Matias [15]	2023	77	9	IA	Endometrioid ca	−	Abdominal pain	S	N/A	HAR	TC + radiation	−	18 alive
Li [8]	2023	68	5	IA	Endometrioid ca	Radiation	Bleeding	R	1.8	LAR	Chemo	−	6 alive
Koury [16]	2021	67	15	N/A	Endometrioid ca	Radiation	Bleeding	S	4	HAR	−	−	12 alive
Jauregui [6]	2021	89	0.1	IIIB	Endometrioid ca	−	Bleeding	S	N/A	−	−	−	N/A
Cardella [17]	2018	77	7	II	Endometrioid ca	Radiation	Bleeding	S	3	HAR	TC	−	8 alive
Hurbers [7]	2017	75	3	IB	Endometrioid ca	Radiation	Abdominal pain	S, small bowel	1.8	LAR	TC	+	6 alive
Molnar [18]^)^	2013	71	2	IIIB	Serous ca	Chemo + radiation	Abdominal pain	A, S, stomach	N/A	Total colectomy	N/A	+	N/A
Anstadt [9]	2012	70	1	IB	Endometrioid ca	−	Bleeding	S	N/A	−	FOLFOX	−	10 recurrence

*A* ascending colon, *Ca* carcinoma, *Chemo* chemotherapy, *FOLFOX* fluorouracil/leucovorin/ oxaliplatin regimen, *HAR* high anterior resection, *LAR* low anterior resection, *LHC* left hemicolectomy, *N/A* not available, *S* sigmoid colon, *T* transverse colon, *TC* paclitaxel/ carboplatin regimen, *R* rectum

## Conclusions

We present a rare case of serous endometrial carcinoma that metastasized to the sigmoid colon, initially mimicking a primary colon carcinoma detected through bowel obstruction. Immunohistochemical studies are essential for an accurate differential diagnosis. Although uncommon, the possibility of metastatic colorectal tumors should be considered in patients with a history of EC, even if several years have passed since the initial surgery.

## Data Availability

The data that support the findings of this study are available from the corresponding author upon reasonable request.

## Abbreviations

CT: Computed tomography
EC: Endometrial carcinoma
PET: Positron emission tomography
